# Supplementary material for: Molecular Subtypes, Metastatic Pattern and Patient Age in Breast Cancer: An Analysis of Italian Network of Cancer Registries (AIRTUM) Data
Source: J Clin Med. 2021 Dec 14;10(24):5873. doi: 10.3390/jcm10245873 (PMC8706111; doi:10.3390/jcm10245873)
Supplement: Supplementary file 1 [file jcm-10-05873-s001.zip › jcm-1453542-supplementary.pdf]

Table S1. AIRTUM 17 population-based registries by year of incidence, number of cases and age standardized rate.

CR*	Incidence Year	n of cases	ASR(W)
Aosta	2011	112	82.7
Bolzano	2011	343	75.8
Sondrio	2011	153	86.3
Varese	2011	778	89.2
Pavia	2011	571	96.3
Genova	2011	873	84.4
Piacenza	2011	256	83.2
Parma	2011	426	95.5
Modena	2011	664	98.5
Reggio Emilia	2012	483	95.0
Toscana	2010	1083	89.4
Brindisi	2010	299	83.9
Catania Messina Enna	2011	1373	79.1
Palermo	2011	789	71.4
Ragusa	2011	173	63.5
Siracusa	2011	294	82.2
Nuoro	2011	161	73.4

*Cancer Registry

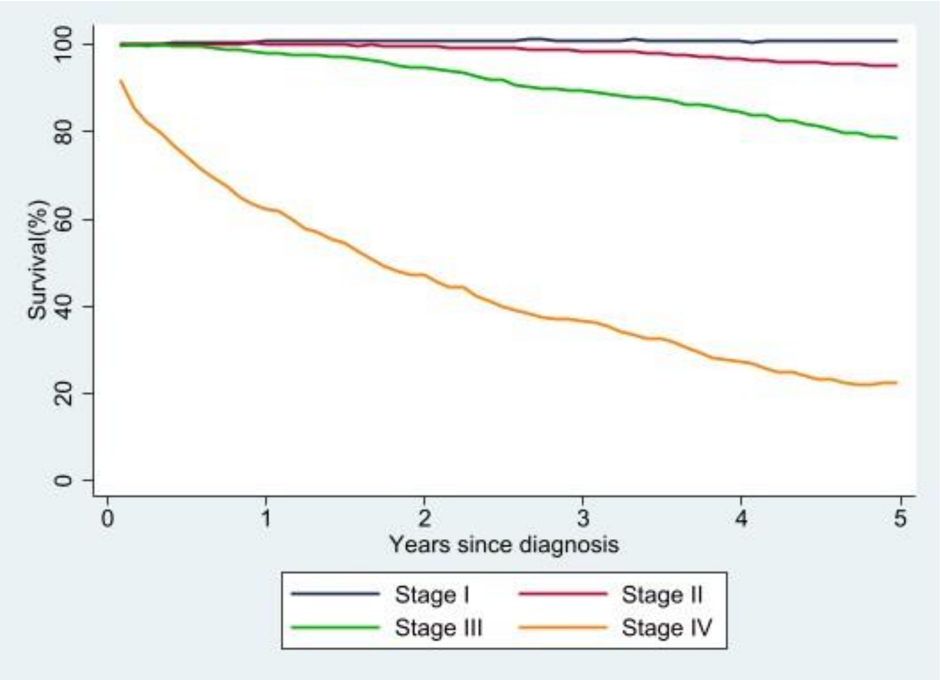

Figure S1. AIRTUM Relative survival by breast cancer stage.
